# *Bothrops atrox* and *Bothrops lanceolatus* Venoms In Vitro Investigation: Composition, Procoagulant Effects, Co-Factor Dependency, and Correction Using Antivenoms

**DOI:** 10.3390/toxins15100614

**Published:** 2023-10-16

**Authors:** Sébastien Larréché, Aurore Bousquet, Lucie Chevillard, Rabah Gahoual, Georges Jourdi, Anne-Laure Dupart, Christilla Bachelot-Loza, Pascale Gaussem, Virginie Siguret, Jean-Philippe Chippaux, Bruno Mégarbane

**Affiliations:** 1 Inserm, UMRS-1144, Université Paris Cité, F-75006 Paris, France; lucie.chevillard@u-paris.fr; 2 Department of Medical Biology, Bégin Military Teaching Hospital, F-94160 Saint-Mandé, France; aurorebousquet@yahoo.fr (A.B.); annelaure.dupart@gmail.com (A.-L.D.); 3 Chemical and Biological Technologies for Health Unit, CNRS UMR 8258, Inserm, Université Paris Cité, F-75006 Paris, France; rabah.gahoual@parisdescartes.fr; 4 Innovative Therapies in Hemostasis, Inserm, Université Paris Cité, F-75006 Paris, France; georges.jourdi@aphp.fr (G.J.); christilla.bachelot-loza@parisdescartes.fr (C.B.-L.); pascale.gaussem@aphp.fr (P.G.); virginie.siguret@aphp.fr (V.S.); 5 Department of Biological Hematology, Lariboisière Hospital, Assistance Publique–Hôpitaux de Paris, F-75010 Paris, France; 6 Department of Hematology, Georges Pompidou European Hospital, Assistance Publique–Hôpitaux de Paris, F-75015 Paris, France; 7 French National Research Institute for Sustainable Development, Université Paris Cité, F-75006 Paris, France; jean-philippe.chippaux@ird.fr; 8 Department of Medical and Toxicological Critical Care, Federation of Toxicology, Lariboisière Hospital, Assistance Publique–Hôpitaux de Paris, F-75010 Paris, France

**Keywords:** *Bothrops*, coagulation, venom composition, ROTEM, co-factor, antivenom

## Abstract

*Bothrops* venoms are rich in enzymes acting on platelets and coagulation. This action is dependent on two major co-factors, i.e., calcium and phospholipids, while antivenoms variably neutralize venom-related coagulopathy effects. Our aims were (i) to describe the composition of *B. atrox* and *B. lanceolatus* venoms; (ii) to study their activity on the whole blood using rotational thromboelastometry (ROTEM); (iii) to evaluate the contribution of calcium and phospholipids in their activity; and (iv) to compare the effectiveness of four antivenoms (Bothrofav^™^, Inoserp^™^ South America, Antivipmyn^™^ TRI, and PoliVal-ICP^™^) on the procoagulant activity of these two venoms. Venom composition was comparable. Both venoms exhibited hypercoagulant effects. *B. lanceolatus* venom was completely dependent on calcium but less dependent on phospholipids than *B. atrox* venom to induce in vitro coagulation. The four antivenoms neutralized the procoagulant activity of the two venoms; however, with quantitative differences. Bothrofav^™^ was more effective against both venoms than the three other antivenoms. The relatively similar venom-induced effects in vitro were unexpected considering the opposite clinical manifestations resulting from envenomation (i.e., systemic bleeding with *B. atrox* and thrombosis with *B. lanceolatus*). In vivo studies are warranted to better understand the pathophysiology of systemic bleeding and thrombosis associated with *Bothrops* bites.

## 1. Introduction

Morbidities and mortality attributed to snakebites represent one of the most threatening plagues in the tropics [1]. In 2017, the World Health Organization (WHO) included snakebite in the list of neglected tropical diseases [2]. Intense attention was drawn with the establishment of an international resolution on snakebites and the proposal of a global strategy for prevention and control [3,4]. The worldwide burden of snakebites has been estimated at ~5 million people per year, with 1.8–2.7 million envenomations and 81–138,000 fatalities [5]. Snake envenoming may result in local edema and necrosis, bleeding and incoagulability, neurotoxicity leading sometimes to respiratory paralysis, acute kidney injury, myotoxicity, cardiotoxicity, hypotension, and thrombosis [6].

*Bothrops* are responsible for the highest number of human envenomations in the Neotropical Americas [7,8,9,10,11,12,13,14,15,16,17]. In the French Departments of America (FDA), *Bothrops atrox* is the predominant species involved in envenomation in French Guiana [18,19], while *B. lanceolatus* is the only venomous snake present in Martinique, where it is endemic [20]. *B. atrox stricto sensu* (named as “*B. atrox*” in the text) and *B. lanceolatus* are phylogenetically close and belong to the *B. atrox* group [21,22]. *B. atrox* and *B. lanceolatus* venoms include snake venom metalloproteinases (SVMPs), phospholipases A_2_ (PLA_2_s), snake venom serine proteinases (SVSPs), C-type lectin-like toxins (CTLs), desintegrins, cysteine-rich secretory proteins (CRISPs), L-amino acid oxidases (LAAOs), and natriuretic peptides consisting in vasoactive peptides and bradykinin potentiating and inhibitory peptides [23]. Composition of *B. atrox* specimens present in French Guiana has never been studied.

*B. atrox* envenomation leads to the typical bothropic syndrome, which combines pain, edema, bruising, blistering, dermo- and myonecrosis, local and systemic bleeding, incoagulability state, circulatory shock, and acute kidney injury [24]. Incoagulability is due to a complex pathophysiology including thrombocytopenia, platelet hypoaggregation, consumption coagulopathy, and fibrin(ogen)olysis [25]. Consumptive coagulopathy is primarily related to the procoagulant venom activity. The procoagulant activity of *Bothrops* venoms was first reported in 1937 by Eagle, who showed that venoms of *B. atrox* and *B. jararaca* converted prothrombin to thrombin, without needing calcium and phospholipids [26]. Even a very low *B. atrox* venom concentration was sufficient to result in a procoagulant activity [26].

Activation of prothrombin by *B. atrox* venom is due to a SVMP [27], while other SVMPs can activate factor X [28,29]. Thrombocytin, an SVSP, also contributes to the procoagulant process by activating factors V and VIII [30]. Other enzymes have anticoagulant activities by directly consuming fibrinogen. Thrombin-like enzymes (TLEs) such as batroxobin cleave fibrinogen into fibrin and induce in vitro clotting of fibrinogen solution leading to friable clots without crosslinking of fibrin [31]. Proteinases having fibrin(ogen)olytic activity may contribute to fibrinogen consumption without conversion to fibrin. Batx-I, from Colombian *B. atrox* venom, is a P-I SVMP degrading preferentially the Aα- and Bβ-chains of fibrinogen, but also inducing a partial degradation of the γ-chain [32].

The first studies on *B. lanceolatus* venom showed no procoagulant activity, as the venom was unable to clot citrated human plasma [33,34,35]. Identification of a thrombin-like enzyme and non-coagulant fibrin(ogen)olytic PLA_2_s and SVMPs suggested a purely anticoagulant activity of the venom [35,36,37]. These initial experimental results were unexpected since *B. lanceolatus* envenomation is unusually associated with systemic bleeding and absence of coagulability, but rather with thrombotic complications such as ischemic stroke, myocardial infarction and pulmonary embolism [38]. One thromboelastographic study performed using human plasma demonstrated the procoagulant potency of *B. lanceolatus* venom [39]. Thromboelastography (TEG) and rotational thromboelastometry (ROTEM) are point-of-care viscoelastic tests of hemostasis. Their major interest is the global assessment of clot formation and dissolution in real time as they use whole blood [40]. While the procoagulant activity of *Bothrops* venom was studied in plasma using TEG [39,41,42,43,44] and ROTEM [45], no in vitro study has been previously conducted in whole blood. Such investigations appear mandatory to understanding the exact venom-induced alterations in fibrinogen/platelet interactions. The activity of *B. lanceolatus* venom on coagulation was confirmed in another study using a kinetic absorbance-based coagulation assay in recalcified plasma [46]. By contrast to early studies, this investigation performed in the presence of calcium, showed that *B. lanceolatus* venom was highly dependent on this co-factor. An additional study performed with *B. atrox* venoms obtained from different Brazil localities confirmed the relative dependency of these venoms on calcium and phospholipids [44].

Bothrofav^™^, a monospecific antivenom, is successfully used to treat *B. lanceolatus* envenomation in Martinique [38,47,48]. Usually, thrombotic complications are not observed when Bothrofav^™^ is started up to 6 h after the bite [38,47]. In French Guiana, the polyvalent antivenom Antivipmyn^™^ TRI (Instituto Bioclon, Mexico, Mexico) is the only antivenom authorized by the French authorities [49], although its effectiveness to correct venom-induced coagulopathy remains controversial. A study conducted at Cayenne University Hospital reported faster normalization in patients who received the antivenom in comparison to the historical group [19]. Another study conducted at the Hospital of Saint-Laurent du Maroni did not find significant effects with this antivenom on the time required to correct hemostasis parameters. Contradictory results were explained by differences in study designs and inter-batch variability, but discrepancies strongly supported the requirement of preclinical studies comparing Antivipmyn^™^ TRI to other available antivenoms before any recommendation on the most suitable product to assess in a clinical trial.

We therefore designed this experimental study aiming (i) to describe the composition of *B. atrox* and *B. lanceolatus* venoms; (ii) to study the activity of these two venoms in whole blood using ROTEM; (iii) to evaluate their dependency on co-factors (i.e., calcium and phospholipids); and (iv) to determine the effectiveness of four antivenoms on their attributed procoagulant activity.

Our findings showed the similar composition of *B. atrox* and *B. lanceolatus* venoms, with a strong procoagulant potency and marked hypercoagulability effects. *B. lanceolatus* venom was completely dependent on calcium to induce activation of plasma coagulation in vitro but less dependent on phospholipids than *B. atrox* venom. The four tested antivenoms were able to neutralize the coagulant activity of the two venoms, although quantitative differences were observed. Bothrofav^™^ showed a greater effectiveness in comparison to the three other antivenoms in neutralizing the procoagulant activity of the venoms.

## 2. Results

### 2.1. Bottom-Up Proteomics Analysis of Venoms

**Figure 1:**
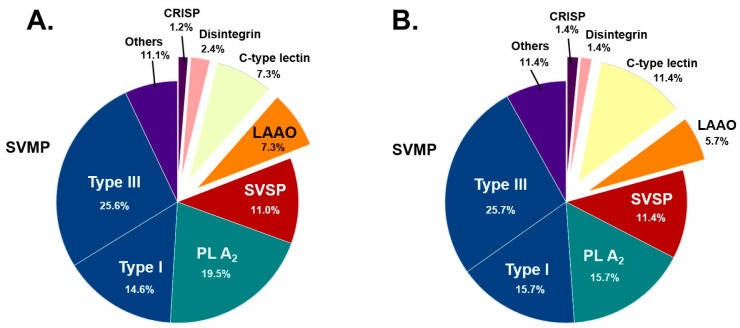
Protein composition of *B. atrox* (**A**) and *B. lanceolatus* (**B**) venoms. SVMP, snake venom metalloproteinase; SVSP, snake venom serine protease; PLA_2_, phospholipases A2, LAAO, L-amino acid oxidase; CRISP, cysteine-rich secretory protein.

**Figure 2:**
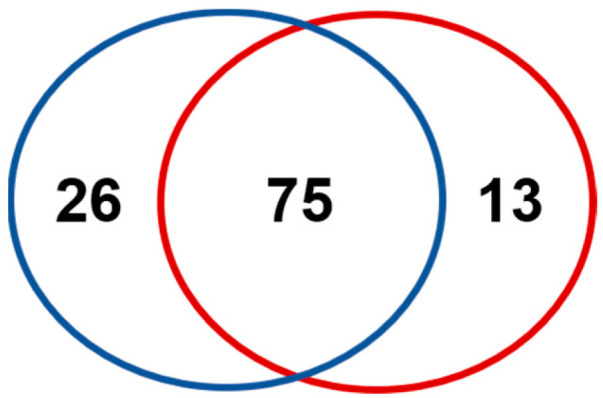
Venn diagram representing the protein diversity identified based on a UPLC-MS/MS approach of *B. atrox* (blue) *and B. lanceolatus* venoms (red).

The protein composition of *B. atrox* and *B. lanceolatus* venoms is presented in Figure 1. In total, ultraperformance liquid chromatography hyphenated to tandem mass spectrometry (UPLC-MS/MS) experiments allowed the identification of 101 distinctive proteins composing *B. atrox* venom and 88 proteins in the case of *B. lanceolatus*. Venom proteins were identified using peptide characterization, concomitantly based on high-resolution *m*/*z* measurement and MS/MS fragmentation attribution [50]. To address the complexity of the venoms, UPLC separation conditions were tailored to maximize the number of detected peptides. In addition, the samples were not submitted to particular fractionation procedures prior to UPLC-MS/MS analysis to prevent protein loss, which might have occurred during sample preparation. Bottom-up proteomics analysis showed that proteins were diverse but distributed in only eight families (Figure 1). SVMPs were the predominant family, representing 40.2% of *B. atrox* venom proteins and 41.4% of *B. lanceolatus* proteins, followed by PLA_2_s (19.51% and 15.71%) and SVSPs (10.98% and 11.43%) as emphasized in Table 1. Most proteins were common to the two venoms, therefore representing 74.2% of *B. atrox* venom and 85.2% of *B. lanceolatus* venom proteins (Figure 2). Proteins identified solely in a single venom type were mainly PLA_2S_ and SVMPs in *B. atrox* venom with 9 and 8 out of 26 proteins, respectively. In *B. lanceolatus* venom, PLA_2S,_ like SVMPs, contained 3 additional proteins.

### 2.2. Rotational Thromboelastometry (ROTEM)

*B. atrox* and *B. lanceolatus* venoms showed marked effects on ROTEM parameters, most of the time without significant differences between the venoms (Table 2). They presented a procoagulant activity in a dose-dependent manner. The clotting time (CT) decreased as venom concentration increased, with a statistically significant effect except at the lowest concentration (10 ng/mL) with both venoms. Of note, the CT for *B. lanceolatus* venom was more prolonged at 100 µg/mL vs. 10 µg/mL. The clot formation time (CFT) also gradually decreased with increasing venom concentration up to a concentration of 1 µg/mL. CFT was longer than the control when *B. atrox* venom was at 100 µg/mL but similar to the control for *B. lanceolatus* venom at 100 µg/mL.

The alpha angle increased slightly with increasing venom concentration up to 1 µg/mL then decreased with higher venom concentrations. The maximal clot firmness (MCF) was most often increased, with a trend to significance. By contrast, the lysis index at 30 min following CT (LI30) was not altered by the venoms. At 100 µg/mL, these two former parameters were significantly reduced.

**Figure 3:**
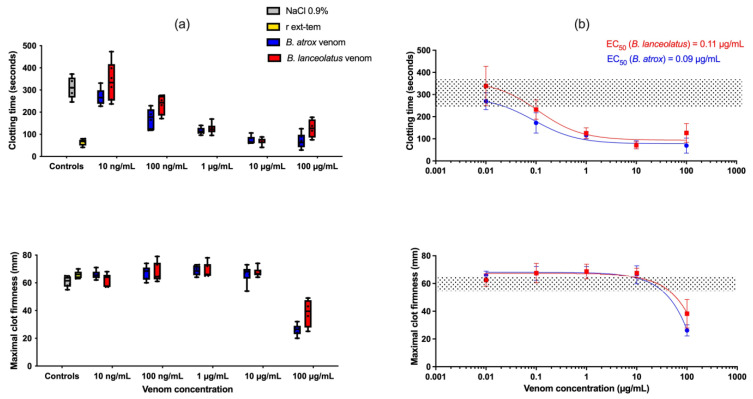
(**a**) Clotting time (CT) and maximal clot firmness (MCF) assessed by ROTEM in human whole blood with 0.9% NaCl (grey boxplots), r ext-tem (recombinant tissue factor and phospholipids used as the positive control, yellow boxplots), *B. atrox* (blue boxplots) or *B. lanceolatus* (red boxplots) venoms; (**b**) Curves representing the CT and the MCF as a function of the dose of *B. atrox* (red line) or *B. lanceolatus* (blue line) venom. Grey dotted area represents the value of the negative control.

Figure 3 shows the distribution of measured values as boxplots for CT and MCF (Figure 3a). The median effective concentration (EC_50_) values for CT decrease were 0.09 and 0.11 µg/mL for *B. atrox* and *B. lanceolatus*, respectively, while EC_50_ for MCF was not calculable (Figure 3b).

### 2.3. Phospholipid and Calcium Dependency

**Figure 4:**
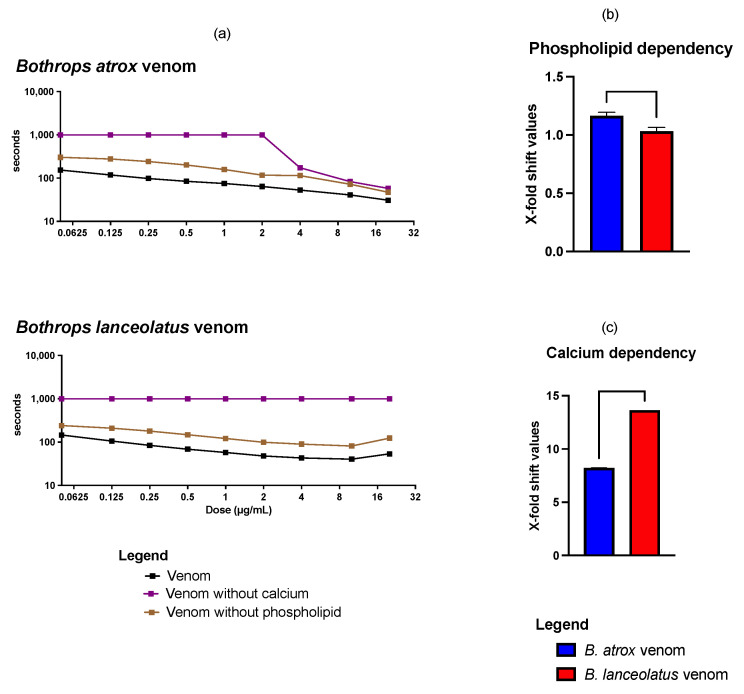
(**a**) Human plasma-clotting times (*y*-axis) induced by *Bothrops* venom with phospholipids and calcium (black line), without calcium (purple line), and without phospholipids (brown line) using nine different venom concentrations (0.05, 0.125, 0.25, 0.5, 1, 2, 4, 10, and 20 µg/mL; *x*-axis). The graph axes are displayed in a logarithmic scale. Each of the nine data points per venom curve is represented by dots (mean value, *n* = 3), with standard deviation error bars but error bars are smaller than the data-point symbol. The grey line represents the value of the negative controls (spontaneous clotting time of plasma with phospholipids and calcium); (**b**) Phospholipid dependency represented by X-fold shift in plasma-clotting time curves. X-fold shift values were calculated by dividing the area under the curve (AUC) for the venom incubated without phospholipid clotting time curve by the AUC for the venom incubated with phospholipids and the calcium clotting time curve and subtracting 1 from the total; (**c**) Calcium dependency represented by X-fold shift in plasma-clotting time curves. X-fold shift values were calculated by dividing the AUC for the venom incubated without the calcium clotting time curve by the AUC for the venom incubated with phospholipids and the calcium clotting time curve and subtracting 1 from the total. Larger numbers indicate greater dependency while smaller values suggest limited dependency. Values are mean (*n* = 3) ± standard deviation.

*B. lanceolatus* and *B. atrox* venoms presented a procoagulant activity in a dose-dependent manner using a platelet-poor plasma (PPP) approach. Areas under the curve (AUC) were determined to assess the procoagulant effects (the more procoagulant the venom, the lower the AUC). *B. atrox* venom had a less procoagulant effect than *B. lanceolatus* venom (626.4 ± 22.75 vs. 546.5 ± 11.76; *p* = 0.0057) (Figure 4). In the absence of phospholipids, venoms had a less significant procoagulant effect than venoms in the presence of calcium and phospholipids, but this effect persisted even at the lowest venom concentration without phospholipids (Figure 4a). The X-fold shift value representing phospholipid dependency was higher for *B. atrox* (1.16 ± 0.03) than for *B. lanceolatus* (1.03 ± 0.03; *p* = 0.0062). In the absence of calcium, the procoagulant effect was observed only from a concentration of 4 µg/mL with *B. atrox* venom while it was never detected with *B. lanceolatus* venom, even at the highest concentration tested (20 µg/mL), and the time machine remained superior to 999 s (Figure 4). The X-fold shift value representing calcium dependency was higher for *B. lanceolatus* (>13.6 ± 0.0) than *B. atrox* (8.2 ± 0.004; *p* < 0.0001). The negative controls (spontaneous clotting time of plasma with both phopholipids and calcium, without calcium and without phospholipids, *n* = 3) were 337.8 ± 7.5 s, >999 ± 0.0 s, and 632.9 ± 14.42 s. The positive controls (clotting time of plasma in the presence of kaolin with phopholipids and calcium, without calcium and without phospholipids, *n* = 3) were 123.4 ± 2.6 s, >999 ± 0.0 s, and 259.5 ± 4.6 s, respectively.

### 2.4. Antivenom Neutralisation

**Figure 5:**
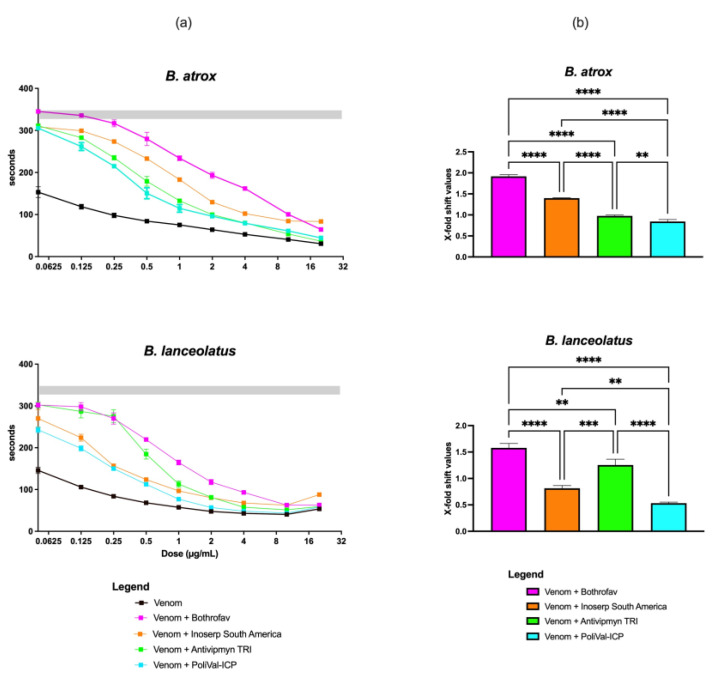
(**a**) Human plasma-clotting times (*y*-axis) induced by *Bothrops* venom without (black line), or with four different antivenoms (line colors indicated by the legend) using nine different venom concentrations (0.05, 0.125, 0.25, 0.5, 1, 2, 4, 10, and 20 µg/mL; *x*-axis). The *x*-axis is displayed in logarithmic scale. Each of the nine data points per venom curve is represented by dots (mean value, *n* = 3), with standard deviation error bars. Some error bars are smaller than the data-point symbols. The grey area represents the value of the negative control (spontaneous clotting time of plasma with both phospholipids and calcium, mean (*n* = 3) ± standard deviation), the grey area represents the value of the negative control; (**b**) X-fold shift in plasma clotting time curves of *Bothrops* venom incubated with four different antivenoms (bar colors indicated by the legend). X-fold shift values were calculated for each antivenom by dividing the area under the curve (AUC) for the venom incubated with the antivenom clotting time curve by the AUC for the venom clotting time curve, and subtracting 1 from the total. Calculated values represent antivenom neutralization: a value of 0 indicates no neutralization, while a value of above 0 indicates neutralization. Values are mean (*n* = 3) ± standard deviation. Tukey’s multiple comparisons tests were used, following significant ordinary one-way analyses of variance (ANOVA). ** *p* < 0.01, *** *p* < 0.001, **** *p* < 0.0001.

Antivenom neutralization was calculated by dividing the AUC for the venom and antivenom curve by the AUC without antivenom and presented as an X-fold shift in the AUC. For B. atrox, the most potent antivenom was Bothrofav^™^ (1.92 ± 0.04), then Inoserp^™^ South America (1.398 ± 0.008), Antivipmyn^™^ TRI (0.98 ± 0.02), and PoliVal-ICP^™^ (0.84 ± 0.05) (Figure 5). For *B. lanceolatus*, the most potent antivenom was Bothrofav^™^ (1.58 ± 0.08), then Antivipmyn^™^ TRI (1.25 ± 0.11), Inoserp^™^ South America (0.81 ± 0.05), and PoliVal-ICP^™^ (0.53 ± 0.08) (Figure 5). By comparing the neutralizing effects obtained with the different venoms using one determined antivenom, Bothrofav^™^, Inoserp^™^ South America, and PoliVal-ICP^™^ had a greater neutralizing effect on *B. atrox* venom than on *B. lanceolatus* venom while Antivipmyn^™^ TRI had a greater neutralizing effect on *B. lanceolatus* venom than on *B. atrox* venom (Figure 5, Table 3).

Antivenoms had no significant effects on clotting as the antivenom controls [Bothrofav^™^ = 349.8 ± 9.5 s, Inoserp^™^ South America = 337.8 ± 7.5 s, Antivipmyn^™^ TRI = 329.0 ± 18.9 s, and PoliVal-ICP^™^ = 317.4 ± 14.9 s] did not significantly differ from the spontaneous CT [F(4.00, 6.37) = 2.765, *p* = 0.12, Brown–Fosythe one-way analysis of variance (ANOVA) and F(4.00, 4.893) = 1.955, *p* = 0.24, Welch ANOVA].

## 3. Discussion

Although responsible for different clinical effects, *B. atrox* and *B. lanceolatus* venoms have comparable proteomes and procoagulant activity. Both proteomes had a predominance of SVMPs. Our findings confirm published data usually reporting a predominance of PI-SVMP and PIII-SVMP [34,51,52,53,54,55]. As has been demonstrated for many venomous species, there is significant individual variability in venom protein composition [56]. Consequently, many factors may be involved in the composition of *B. atrox* venom, such as gender [57], geographical distribution [58], habitat [59], ontogeny [60,61], and captivity [62]. Gender-based variation in *B. atrox* venom was observed even in siblings [63]. Venoms from newborn and juvenile specimens showed higher hemorrhagic and procoagulant activities, when compared with venoms obtained from 3-year-old snakes [64]. Envenoming by adult *B. atrox* snakes causes more severe local inflammatory effects, whereas venom-induced coagulopathy is more frequent in envenoming by juvenile specimens [65]. No study is available on the variability factors of *B. lanceolatus* venom.

Consistent with SVMP predominance, our ROTEM study found a procoagulant activity for both *B. atrox* and *B. lanceolatus* venoms illustrated by a decrease in CT inversely proportional to venom concentration, confirming previous studies [39,44,46,66,67]. Since CT is essentially linked to the concentration of clotting factors [68], our data suggest the presence of factor activators in the two venoms. SVMPs, which can activate prothrombin and factor X, have already been isolated in *B. atrox* venom [27,28,29] but not yet in *B. lanceolatus* venom. ROTEM analysis showed greater procoagulant activity of *B. atrox* venom, although not statistically significant, while coagulation time evaluation in human PPP found *B. lanceolatus* venom to be more powerful. However, using *B. lanceolatus* venom at 100 µg/mL, a paradoxical increase in CT was observed. This result suggested instantaneous consumption of a part of the clotting factors due to an activator present in this venom and, therefore, a procoagulant activity greater than that of *B. atrox* venom at this concentration. Using a protocol similar to ours, Bourke et al. found a coagulation time of 208.3 ± 7.6 s with 20 µg/mL of *B. lanceolatus* venom, whereas 20 µg/mL of *B. atrox* venom from French Guiana had a coagulation time of 88.3 ± 2.9 s [39]. Interestingly, this study found a very short coagulation time (38.3 ± 2.9 s) with the venom of *B. caribbaeus*, the phylogenetically closest species to *B. lanceolatus* [39]. Another study comparing coagulation times of different *Bothrops* finds shorter values for *B. lanceolatus* than *B. atrox* from Colombia [46]. Of note, *B. atrox* from French Guiana was not evaluated. These two venoms seem to have comparable procoagulant effects, without significantly different EC_50_ using ROTEM.

The decrease in CFT and increase in alpha angle reflecting fibrinogen level and fibrin polymerization [68] highlight both thrombin generation by factor activators and the activity of thrombin-like enzymes isolated from *B. atrox* and *B. lanceolatus* venoms. For the lowest concentrations (from 10 ng/mL to 1 µg/mL), CFT gradually decreased while alpha angle increased, which corresponds to increasingly rapid formation of fibrin under the influence of thrombin-like enzymes. For higher concentrations (10 and 100 µg/mL), CFT increased and alpha angle decreased, further reflecting the consumption of fibrinogen by thrombin-like enzymes. At 100 µg/mL, CFT increased and alpha angle decreased more markedly with *B. atrox* than *B. lanceolatus* venom. This difference may illustrate a greater thrombin-like activity of *B. atrox* venom at this concentration. MCF was increased with the two venoms, but not consistently with statistical significance. Previous investigations carried out using TEG in plasma found a decreased amplitude of the clot [39,44]. In reality, these plasma-based studies only revealed fibrinogen consumption, whereas ROTEM MCF using whole blood evaluated both fibrin formation and platelet activity [68], explaining why it enabled highlighting the hypercoagulability of the two venoms. MCF did not seem very sensitive to the venom effects. The two phenomena induced by venoms might also cancel each other out: activation of fibrin polymerization, which strengthened the clot and consumption of fibrinogen, which reduced it. However, at 100 µg/mL, consumption became greater, and the amplitude of the clot decreased significantly. Thrombocytin isolated from *B. atrox* venom induced platelet-aggregation with a release of its content [69]. However, a direct activating effect of venom is unlikely since whole venom did not induce aggregation of washed rabbit platelets [70]. For *B. lanceolatus* venom, no direct effect on platelet aggregation was observed whereas it inhibited collagen-induced platelet aggregation [34]. This hypercoagulability could be due to an indirect effect of the venom, by increasing thrombin generation, activating factor XIII and/or inducing cytokine release and thromboinflammation. Brazilian *Bothrops* venoms (i.e., *B. moojeni*, *B. jararacussu*, and *B. alternatus*) increased in vitro values of endogenous thrombin potential of human PPP, even without the addition of tissue factor as a trigger [71]. Thrombocytin is able to activate factor XIII by limiting its proteolysis and to increase the procoagulant activity of factor VIII in a manner analogous to that of thrombin [30]. Once bound to fibrin, the capacity of batroxobin, a TLE from *B. atrox* venom, to promote fibrin accretion was found to be 18-fold greater than that of thrombin [72]. In an ex vivo human whole blood model, *B. lanceolatus* venom elicited an inflammatory reaction, with pro-inflammatory interleukin production, chemokine upregulation, complement activation, and eicosanoid release [73]. Increases in interleukin-1β, interleukin-6, and monocyte chemoattractant protein-1 were additionally observed in patients bitten by *B. atrox* [74,75].

The fibrinolytic activity of the venoms seems limited in view of the absence of modification of LI30 for most concentrations tested. With the highest concentrations, the sudden consumption of coagulation factors and fibrinogen and hyperfibrinolysis result in an increase in CT and CFT and a reduction in alpha angle, MCF, and LI30. The same paradoxical effect was described in vitro with *B. lanceolatus* venom. At low concentrations, the venom induces a clot on purified fibrinogen by its thrombin-like activity, whereas at high concentrations, no clot is observed due to the instantaneous action of fibrinogenases [35].

Calcium ions and phospholipids are key players of the coagulation pathway. For instance, in an in vitro kinetic analysis, calcium stimulated the activation of factor X by activated factor VII 10-fold then in the presence of calcium, phospholipids caused a 2-fold increase in the apparent velocity of the reaction [76]. Although it can clot plasma in the absence of calcium and phospholipids, *B. atrox* venom was shown to be more effective in the presence of these two cofactors. For this species, dependency on phospholipids was more important than dependency on calcium. The factor X activator isolated by Hofmann et al. was calcium-dependent [28]. A significant variation in dependency on calcium and phospholipids was suggested for Brazilian *B. atrox* from different localities, with the assessment of correlation between dependency to the different co-factors but without correlations with the overall procoagulant activity or relative factor X or prothrombin activation activities [59]. The *B. atrox* venom used in our experiments is a pool from different localities (French Guiana, Peru, Brazil). Therefore, the variability of the dependence on co-factors depending on the geographical origin could not be assessed in this study.

*B. lanceolatus* venom is totally dependent on calcium and to a lesser extent on phospholipids, as we showed, to the best of our knowledge, for the first time. In contrast, the total dependency on calcium was already reported with other venoms including those of Australian *Elapidae including Cryptophis nigrescens*, *Demansia papuensis*, *D. psammophis*, *D. vestigiata*, *Hemiaspis damelii*, *H. signata*, *Pseudecis prophyriacus*, *Suta punctata* [77], and *Echis coloratus* from Saudi Arabia [78]. This is probably also the case for *B. caribbaeus*, which is not able to clot plasma in the absence of calcium [34], although this finding requires confirmation. For the genera *Oxyuranus*, *Pseudonaja*, and *Notechis*, the less calcium and phospholipid-dependent venoms were also shown to be the more potent venoms [79,80]. Similarly, some *Pseudonaja* venoms induced faster clotting times in the absence of phospholipids [80]. Finally, calcium and phospholipids had no impact on the clotting time in the presence of venoms of *Dispholidus typus* and *Thelotornis mossambicanus*, two African venomous Colubridae [81].

The four tested antivenoms were able to neutralize the coagulant activity of the two *Bothrops* venoms, albeit with quantitative differences, consistent with previously published data showing a high degree of cross-neutralization of bothropic and polyspecific antivenoms manufactured in Latin America against a variety of *Bothrops* venoms [82]. Bothrofav^™^ was the antivenom that showed the best efficacy on coagulopathy induced by these venoms. These findings support the similarities in venom composition. This monovalent antivenom has already demonstrated its effectiveness in *B. lanceolatus* envenomation in Martinique [38,47,48]. However, no clinical data regarding *B. atrox* envenomation treated using Bothrofav^™^ are available. The first study on neutralization of *B. atrox* venom from Colombia using Bothrofav^™^ showed that this antivenom was effective in reversing the lethal, hemorrhagic, myotoxic, and indirect hemolytic effects, but only partially active in neutralizing edema-forming activity [46]. In contrast, Bothrofav^™^ was ineffective against coagulopathy and fibrinogenolytic activities. More recently, Bourke et al., whose methodology was reproduced in our experimental work, showed that Soro Antibotrópico^™^ (Instituto Butantan, Sao Paulo, Brazil) and Bothrofav^™^ were the two most effective antivenoms to limit the coagulopathy activity of *B. atrox* from French Guiana, ranking before PoliVal-ICP^™^, Antivipmyn^™^ TRI, and Antivipmyn^™^ [66]. Another study showed that Bothrofav^™^ neutralized the coagulopathic activity of *B. atrox* venom from Guyana and Suriname, but not from Colombia, confirming the initial observations [46]. Here, we used a pool of *B. atrox* venom obtained from different localities; thus, precluding identifying differences in the geographical origin-dependent efficacy of the antivenoms.

Inoserp^™^ South America is an antivenom currently in development. Its immunization panel contains *B. atrox* and *B. lanceolatus* venoms unlike the other two polyvalent antivenoms, which perhaps explains its good results against *B. atrox* venom. Compared to Bothrofav^™^, it offers the advantage of being freeze-dried and therefore less dependent on the cold chain than a liquid antivenom. A recent study suggested the benefit of making an antivenom available in peripheral centers in French Guiana, to reduce the administration time, because time from snakebite to the normalization of clotting parameters was shorter in patients receiving antivenom up to 6 h after the snakebite [83]. A freeze-dried antivenom is particularly interesting for physicians practicing in an isolated situation such as military physicians deployed in the setting of the fight against illegal gold panning. Another advantage of this polyvalent antivenom is coverage against species other than *Bothrops* spp. such as *Lachesis muta* or *Crotalus durissus* also present in French Guiana, even though they are responsible for few envenomation cases in this French department.

Antivipmyn^™^ TRI was effective against the two studied venoms. More specifically, it was the most effective polyvalent antivenom against *B. lanceolatus* venom. In the event of a Bothrofav™ stock shortage, Antivipmyn^™^ TRI might thus be used as an alternative, but this strategy still requires a confirmation based on a clinical trial. Antivipmyn^™^ TRI and PoliVal-ICP^™^ were less effective on *B. atrox* venom than Bothrofav^™^ and Inoserp^™^ South America. The first two antivenoms are made from a panel that does not include *B. atrox* venom but *B. asper* venom. Antivenoms produced against Central American *Bothrops* species are less effective in neutralizing the effects of Amazonian *Bothrops* venoms [84]. The lack of effectiveness to reverse coagulopathy, reported by Heckmann et al. in French Guiana [18], might also be due to an insufficient dosage. At that time, the protocol recommended only three vials. This was in contradiction with the manufacturer’s instructions recommending between three and 16 vials depending on severity. In Colombia, the dose regimen of Antivipmyn^™^ TRI is four vials in an envenomation and up to 12 vials in the most severe cases of *B. asper* bites [85].

PoliVal-ICP^™^ was also effective but less than the three other antivenoms. PoliVal-ICP^™^ is an antivenom originally designed for Central American snakes, especially for *B. asper*, and seems less effective for *B. atrox* [66]. A preclinical study compared PoliVal-ICP^™^ and Antivipmyn^™^ TRI against *B. atrox* venom showed a better neutralization of coagulant activity of PoliVal-ICP^™^ [86]. This difference can be explained by the inter-batch variability. At equivalent concentrations, PoliVal-ICP^™^ and Bothrofav^™^ seem to exhibit a similar neutralizing effect on the coagulopathic activity of *B. atrox* venom [46].

Our study has limitations. Our in vitro approach focused on the immediate effect of venoms on hemostasis and, more precisely, on coagulation. This effect is manifested clinically by the absence of coagulability, but bleeding is first linked to hemorrhagic SVMPs, which generate vascular lesions by proteolysis of the basement membrane. Similarly, thrombosis described during *B. lanceolatus* envenomation occurs in a staggered manner. It is therefore necessary to have an additional dynamic approach based on an animal model to identify the hemorrhagic and thrombotic determinants of *Bothrops* envenomation.

To evaluate neutralization by antivenoms, we used a comparative approach at constant venom volumes. Due to the high variability in protein concentration of the evaluated products, it would have been interesting to additionally compare them at an equivalent concentration. However, the antivenom composition (IgG for PoliVal-ICP and F(ab’)2 for the others) and the unknown degree of purity make this approach unreliable. Moreover, we used only one batch for each antivenom. Ideally, the investigations should have been repeated with different batches to consider inter-batch variability, but we did not have the opportunity to do so during our study. It is therefore worth bearing in mind the qualitative efficacy of the tested antivenoms in relation to the coagulopathic effects of the venoms rather than the comparisons between their activities.

Another limitation of this study is that experiments were focused on hemostasis disorders. Bothrops envenomation is a complex disease also causing bleeding, edema, necrosis, shock, and acute kidney injury. However, the efficacy of the tested antivenoms to neutralize the procoagulant activity of the venoms suggests that none should be ruled out at this stage but all evaluated in in vivo studies assessing other damage.

## 4. Conclusions

Consistent with their phylogenetic proximity, *B. atrox* and *B. lanceolatus* possess very similar venoms in terms of composition and effects on hemostasis. Our findings are surprising in view of the opposite clinical pictures resulting from envenomation by these two species. In vivo studies are thus needed to understand the pathophysiology of systemic bleeding and thrombosis associated with *Bothrops* bites. The total dependency on calcium for *B. lanceolatus* venom may explain the absence of procoagulant effects observed in early studies. Our findings on antivenom-based neutralization support the possibility of using a single antivenom in the two French departments of America facing snakebite envenomation, although such a strategy should be validated with an appropriate clinical trial.

## 5. Materials and Methods

### 5.1. Venoms

Freeze-dried venoms were obtained from Latoxan (Valence, France). *B. atrox* venom (batch 211.191) is a pool of samples from 76 snakes: wild-caught or born in captivity, male and female adults, from French Guiana, Peru, and Brazil. *B. lanceolatus* venom (batch 411.171) is a pool of samples from wild-caught snakes from Martinique, two males and one female adult. Venoms were prepared as a 10 mg/mL stock solution in saline phosphate buffer (PBS), aliquoted and stored at −20 °C until experimentation.

### 5.2. Antivenoms

Expired antivenoms were used (Table 4). Inoserp^™^ South America and Antivipmyn^™^ TRI were reconstituted in sterile water while Bothrofav^™^ and PoliVal-ICP^™^ were already in liquid form. The protein concentrations of antivenoms were determined with a Cobas 6000 analyzer system^™^ (Roche Diagnostics, Mannheim, Germany) using a Total Protein Gen.2^™^ reagent (Roche Diagnostics). Antivenoms were aliquoted and stored at +4 °C until experimentation. They were used in experiments between August 2022 and March 2023. Antivenoms retain their effectiveness after their expiration date, which allowed their use in this experimental study [87].

### 5.3. Human Blood

Human whole blood from healthy donors was obtained from the Etablissement Français du Sang (EFS) (C CPSL UNT-N°13/EFS/064) and collected in 4.5 mL BD Vacutainer^™^ tubes containing a solution of 3.2% sodium citrate. Complete blood count was performed on each sample before it was given to us. Whole blood was obtained the day of each ROTEM experiment and used up to 4 h after sampling (*n* = 6).

### 5.4. Human PPP

Human PPP (3.2% citrated) from healthy donors was bought from the Centre de Transfusion Sanguine des Armées (CTSA). Once obtained, the plasma was stored at −80 °C until aliquoted into 3 mL tubes. Platelets, coagulation factors, and fibrinogen were measured to check plasma quality. A residual platelet count was performed on an XN 1000 analyzer (Sysmex, Kobe, Japan) and was undetectable. Coagulation factors and fibrinogen were measured on a STA-R-Max^™^ automated coagulation analyzer system (Stago, Asnières-sur-Seine, France) and the results are presented in Table 5. The plasma was then aliquoted and stored at −80 °C until experimentation. Before each experiment, the plasma was defrosted for 4 min in a 37 °C water bath, vortexed, and then used within 4 h (*n* = 6).

### 5.5. Proteomic Analysis

Snake venom sample tryptic digestion. A volume of 2.5 µL of crude venom originating from *B. atrox* and *B. lanceolatus* at a concentration of 10 mg/mL was sampled and diluted using 8.75 µL milli-Q H_2_O. The sample was then diluted by adding 10 µL of ammonium bicarbonate 50 mmol/L (pH 8.0) and the samples were incubated at 40 °C for 10 min. Dithiotreitol (DTT) provided by Sigma-Aldrich (Saint-Quentin Fallavier, France) was added to the sample to obtain a final concentration of 10 mmol/L. The samples were then heated to 80 °C for 20 min. The sample was then cooled down to room temperature and iodoacetamide (IAM) was added to a final concentration of 10 mmol/L. The samples were then incubated at room temperature for 20 min in the dark to allow alkylation of thiols residues. A volume of 1 µL of trypsin (0.25 µg/µL) was added to the mixtures which were left for incubation at room temperature for 2 h. Another volume of 1 µL was added and digestion was performed overnight at 37 °C. Following digestion, a volume of 1 µL of formic acid (FA) 98% was added and the sample was left at room temperature for 1 h. Finally, the samples were diluted to a final concentration of 0.25 µg/µL using H_2_O (0.1% FA). Digested samples were stored at 5 °C prior to analysis.

Peptide mixture characterization using ultraperformance liquid chromatography–tandem mass spectrometry (UPLC-MS/MS). Peptide mixtures generated from tryptic digestion were separated by reverse phase UPLC (ACQUITY^™^, Waters, Manchester, UK) using a C18 stationary phase (BEH C18 1.7 µm, 2.1 × 150 mm) purchased from Waters (St. Quentin-en-Yvelines, France) directly hyphenated to a LTQ orbitrap XL^™^ mass spectrometer (Thermo Scientific, Bremen, Germany). The mobile phases were composed of 0.1% formic acid (FA) in water (mobile phase A) and 0.1% FA in acetonitrile (mobile phase B). Peptide separation was carried out using a gradient from 5 to 90% B in 180 min and maintained at 90% B for 3 min, at a flowrate of 100 µL/min. The sample volume used for UPLC-MS/MS experiments was systematically 10 µL. LTQ orbitrap XL^™^ MS was equipped with heated electrospray ionization source (HESI-II) from Thermo Scientific (Bremen, Germany). ESI source parameters were set as follows: ESI voltage −4.0 kV, sheath gas flowrate value was 40 and an auxiliary gas flowrate value of 12. ESI nebulizer temperature was set at 300 °C. The capillary voltage and tube lens were set at 35V and 90V, respectively. MS/MS experiments were performed in a Top5 data-dependent acquisition (DDA) composed of one full MS scan over the mass/charge (*m*/*z*) range 150–2000, followed by five sequential MS/MS realized on the five most intense ions detected at a minimum threshold of 500 counts. Full MS scans were collected in profile mode using the high resolution FTMS analyzer (R = 30,000) with a full scan AGC target of 1E6 and microscans = 1. The ion trap was used in centroid mode at a normal scan rate to analyze MS/MS fragments. The MSn AGC target was set to 1E4 with microscans = 3. Ions were selected for MS/MS using an isolation width of 2 Da, then fragmented by collision induced dissociation (CID) using a normalized CID energy of 35, an activation Q of 0.25, and an activation time of 30 ms. The default charge state selected was z = 2. Using these parameters, the total duty cycle was determined to be 0.65 s. Parent ions were excluded from MS/MS experiments for 60 s in case ions triggered an event twice in 15 s using an exclusion mass width of ±1.5 Th. The instruments were controlled using Xcalibur 2.1.0 SP1 Build 1160^™^ (Thermo Scientific, Bremen, Germany).

MS/MS data analysis. Data obtained from the UPLC-MS/MS experiments were manually analyzed when necessary, using Xcalibur Qual Browser 2.2 SP1.48^™^ (Thermo Scientific, Bremen, Germany). Protein identification was performed using SearchGUI^™^ (University of Bergen, Bergen, Norway) software [88] by the intermediate of the MSGFplus™ search algorithm (University of California, CA, USA) [89]. Tryptic peptides were identified using SwissProt^™^ data (UniProtKB/Swiss-Prot Release 2023_02 of 3 May 2023) for the *Bothrops* snake taxonomy. Conventional cleavage rules were applied, carbamidomethylation of cysteine (+57.0215 Th) considered as a systematic modification and methionine oxidation (+15.9959 Da) as a potential modification. Additional peptide identification parameters were maximum missed cleavages: 2, peptide mass tolerance: 5 ppm in MS and MS–MS tolerance: 0.5 Da.

### 5.6. Rotational Thromboelastometry (ROTEM)

The experiment consisted of testing human whole blood by ROTEM after adding different concentrations of venom or r ex-tem (recombinant tissue factor and phospholipids, #503-05, used as the positive control) or PBS (the negative control). The triggering reagent was therefore either venom or r ex-tem while the addition of saline evaluates spontaneous coagulation. Rotational thromboelastometry was performed on a ROTEM Delta^™^ analyzer (Werfen, Le Pré-Saint-Gervais, France). For each venom, the ROTEM was performed at five different venom concentrations (100; 10; 1; 0.1, and 0.01 μg/mL). Venom stock was diluted in PBS to obtain a 3.5 mg/mL solution. On each tube of whole blood (*n* = 6), the controls and all the concentrations corresponding to the two venoms were tested. The citrated whole blood tube was placed in the sample pre-heating station (at 37 °C) of the ROTEM analyzer. For the first venom concentration (100 µg/mL), all reagents were added into the cup at the following volumes: 20 µL CaCl_2_ (Star-tem, #503-01), 20 µL venom sample, and 300 µL whole blood. Viscoelasticity data were then recorded at 37 °C for 60 min. For the other venom concentrations, the volume of venom solution was adjusted to 20 µL using PBS. The positive control consisted of the same procedure with 20 µL of reagent r ex-tem instead of venom solution, whereas the venom solution was replaced with 20 µL of PBS for the negative control (thus corresponding to spontaneous coagulation activation in whole blood). The parameters assessed by ROTEM include CT, CFT, alpha angle, MCF, and LY30. CT is the time (s) from the start of the measurement until the initiation of clotting (i.e., clot firmness of 2 mm above baseline), and depends on clotting factors levels. CFT is the time interval (s) between the initiation of clotting until a clot firmness of 20 mm above baseline, and depends on the fibrinogen level. The alpha angle is the angle (°) of the tangent at 2 mm amplitude, and depends on the fibrinogen level. MCF is the maximum clot firmness (mm) reached during the run, and depends on platelet count and function and on fibrin formation. LI30 is the residual clot firmness at 30 min from CT and represents the fibrinolysis phase [40].

### 5.7. Coagulation Experiments in PPP

The experiment consisted of testing human plasma after adding different concentrations of venom or kaolin (positive control) or Owren–Koller (OK) buffer (negative control). The triggering reagent was therefore either venom or kaolin while the addition of OK buffer evaluates spontaneous coagulation. We used the methodology described by Rogalski et al. to study the procoagulant effect of *Echis* venom [78] for all coagulation experiments in PPP. Coagulation analyses were performed on a STA-R Max^™^ automated coagulation analyzer system (Stago, Asnières sur Seine, France). The reagents used were: OK buffer (STA- Owren–Koller #00360), calcium (STA-CaCl_2_ 0.025 M #00367), and phospholipids (cephalin prepared from rabbit cerebral tissue from STA-C.K. Prest 5 #00597, solubilized in OK buffer). For each venom, the clotting time of PPP was measured in triplicate at nine different venom concentrations (20; 10; 4; 2; 1; 0.5; 0.25; 0.125; and 0.05 μg/mL). Venom stock was diluted 1:100 in OK buffer to obtain a 0.1 mg/mL solution. For the first venom concentration (20 µg/mL), 50 µL of calcium and 50 µL of phospholipids were added to 50 µg/mL of venom solution at 0.1 mg/mL. An additional 25 µL of OK buffer was added to the cuvette of the analyzer, which was incubated for 2 min at 37 °C, before adding 75 µL of PPP into the cuvette (final volume = 250 µL). Time until clot formation was immediately monitored by the automated analyzer according to a chronometric method. For other venom concentrations, the volume of venom solution was adjusted with OK buffer. The positive control consisted of the same procedure with 50 µL of kaolin (from STA-C.K. Prest 5 #00597) instead of venom solution while in the negative control venom solution was replaced with 50 µL of OK.

### 5.8. Investigation of Phospholipid and Calcium Dependency

For each venom, the coagulation analyses were run with and without calcium and/or phospholipids. The experimental protocol and the reagents were identical, with the exception that 50 µL of OK buffer was added instead of calcium or phospholipids, to maintain the same final volume as in previous experiments. Tests were conducted in triplicate. The cofactor dependency was calculated by an X-fold shift value as dividing the AUC for the venom incubated without cofactor clotting time curve by the AUC for the venom incubated with phospholipids and calcium clotting time curve and subtracting 1 from the total. Calculated values represent phospholipids or calcium dependency. Larger numbers indicate greater dependency, whereas a null value corresponds to no change in the presence or absence of a cofactor. Values are expressed as mean ± standard deviation.

### 5.9. Antivenom Neutralization

Each antivenom was diluted 1:20 in OK buffer (50 μL of AV in 950 μL of OK buffer). The conditions of coagulation analysis for the venom were then identically replicated with 25 µL of diluted AV instead of 25 µL of OK buffer, to maintain the same final volume as in previous experiments. Procedures for the negative controls were performed like those in 5.7. to check that antivenom has no intrinsic procoagulant or anticoagulant effect. Tests were conducted in triplicate.

### 5.10. Statistical Analysis

All statistical analyses were performed with GraphPad PRISM 9.5.0^™^ (GraphPad Prism Inc., La Jolia, CA, USA). All results in the study are shown as the mean ± standard deviation. A *p*-value of less than 0.05 was considered significant. When reporting *p*-values and the associated information, the following abbreviations are used: *p* means *p*-value and F means F-value. When reporting F-values, the degrees of freedom are shown in brackets. Data were tested for normality by visual inspection and by the Shapiro–Wilk test.

The *B. atrox* and *B. lanceolatus* venoms were compared with each other then, with saline at each concentration and for each ROTEM parameter using multiple *t*-tests. For parameters with normal distribution, an unpaired Welch *t*-test was performed for each venom concentration. For parameters with non-normal distribution, a Mann–Whitney test was performed for each venom concentration. A two-stage linear step-up procedure of Benjamini, Krieger, and Yekuteli completed the procedure. The distribution of measured values was represented as boxplots for CT and MCF.

Nine-point dilution curves, showing the clotting time of each venom in plasma, with or without calcium, phospholipids, or antivenom, were graphed using GraphPad PRISM 9.5.0^™^. To more clearly view the data, the *x*-axis for venom concentration and for cofactor dependency the *y*-axis for clotting time were presented in logarithmic view.

To assess the procoagulant effect, the AUC for each venom with both calcium and phospholipid was compared using an unpaired *t*-test.

The cofactor dependency was calculated by an X-fold shift value by dividing the AUC for the venom incubated without a calcium or a phospholipid CT curve by the AUC for the same venom incubated with both a phospholipid and a calcium CT curve and subtracting 1 from the total. Larger numbers indicate greater dependency while smaller values suggest limited dependency. If there was no change with or without a cofactor, this would have a value of 0. Within each cofactor, the X-folds shifts between the different venoms were compared using an unpaired *t*-test.

Antivenom neutralization was calculated by an X-fold shift value by dividing the AUC for the venom incubated with antivenom CT curve by the AUC for the same venom alone CT curve and subtracting 1 from the total. A value of 0 indicates no neutralization and a value above 0 indicates neutralization. Within each venom, the X-fold shifts for different antivenoms were also compared using an ordinary ANOVA. The post hoc test used was Tukey’s multiple comparisons test, in which multiplicity adjusted *p*-values were used that accounted for multiple comparisons. The X-fold shifts between different antivenoms for each venom were compared using an unpaired *t*-test. To test if antivenoms had any effect on CT, CTs of the antivenom controls were compared to the spontaneous control using the Brown–Forsythe ANOVA. A Welch ANOVA was also performed alongside due to uncertainty as to which test is best.

## Figures and Tables

**Table 1:** Relative occurrence of different protein families (as a percentage of the total UPLC-MS/MS-identified proteins) in *B. atrox* and *B. lanceolatus* venoms.

Protein Family	% of Total Venom Proteins	
	*Bothrops atrox*	*Bothrops lanceolatus*
Calmodulin	1.22	1.43
C-type lectin	7.32	11.43
Cystatin	1.22	-
Cysteine-rich secretory protein	1.22	1.43
Disintegrin	2.44	1.43
L-amino acid oxidase	7.32	5.71
Natriuretic peptide	2.44	2.86
Nerve venom growth factor	2.44	4.29
Phospholipase A2	19.51	15.71
PI-SVMP	14.63	15.71
PIII-SVMP	25.61	25.71
SVSP	10.98	11.43
Other protein categories	3.65	2.86

**Table 2:** ROTEM parameters for the controls and venom concentrations of *B. atrox* and *B. lanceolatus*. CT: clotting time, CFT: clot formation time, Alpha: alpha angle, MCF: maximal clot firmness, LI30: clot lysis at 30 min following MCF. Values are presented as mean ± SD. *n* = 6 for the controls and venoms, except *B. lanceolatus* 100 µg/mL (*n* = 4). Multiples *t*-tests were used for comparing *B. atrox* and saline (^a^), and *B. lanceolatus* and saline (^b^), and *B. atrox* and *B. lanceolatus* (^c^): Welch *t*-tests for parameters with normal distribution and Mann–Whitney tests for parameters with non-normal distribution.

	Venom Concentration	Saline	Ext-Tem	*B. atrox*	*p*-Value ^a^	*B. lanceolatus*	*p*-Value ^b^	*p*-Value ^c^
CT (seconds)	0 ng/mL	310.8 ± 48.5	63.7 ± 14.6					
	10 ng/mL	-	-	269.6 ± 38.3	0.1644	338.6 ± 88.8	0.5283	0.1112
	100 ng/mL	-	-	171.8 ± 45.7	0.0011	232.3 ± 43.9	0.0229	0.0414
	1 µg/mL	-	-	115.5 ± 15.8	0.0005	125.3 ± 24.4	0.0003	0.4270
	10 µg/mL	-	-	73.0 ± 18.0	0.0002	70.5 ± 16.1	0.0002	0.8049
	100 µg/mL	-	-	69.2 ± 34.2	<0.0001	126.5 ± 42.6	0.0005	0.0458
CFT (seconds)	0 ng/mL	90.5 ± 11.6	58.5 ± 1.8					
	10 ng/mL	-	-	62.3 ± 5.6	0.0022	69.7 ± 15.1	0.0245	0.5667
	100 ng/mL	-	-	61.5 ± 17.8	0.0152	58.2 ± 8.0	0.0003	0.9004
	1 µg/mL	-	-	50.8 ± 10.0	0.0022	49.8 ± 6.8	<0.0001	0.9740
	10 µg/mL	-	-	55.3 ± 6.6	0.0022	67.3 ± 15.8	0.0175	0.1429
	100 µg/mL	-	-	249.3 ± 79.6	0.0022	87.2 ± 24.2	0.8147	0.0095
Alpha (°)	0 ng/mL	72.3 ± 2.0	77.8 ± 1.5					
	10 ng/mL	-	-	77.7 ± 1.2	0.0022	75.5 ± 2.9	0.0538	0.1796
	100 ng/mL	-	-	77.7 ± 3.4	0.013	78.3 ± 1.6	0.0002	0.0649
	1 µg/mL	-	-	79.7 ± 2.0	0.0022	80.3 ± 1.2	<0.0001	0.5887
	10 µg/mL	-	-	78.7 ± 1.5	0.0022	80.2 ± 3.5	0.0014	0.6991
	100 µg/mL	-	-	59.8 ± 10.2	0.0585	74.0 ± 2.9	0.3677	0.0667
MCF (mm)	0 ng/mL	60.8 ± 4.1	66.8 ± 3.5					
	10 ng/mL	-	-	65.8 ± 3.1	0.0455	62.5 ± 4.5	0.6147	0.2446
	100 ng/mL	-	-	67.3 ± 5.0	0.0563	67.5 ± 7.0	0.1429	0.9740
	1 µg/mL	-	-	68.7 ± 3.6	0.087	68.7 ± 5.3	0.0065	0.7056
	10 µg/mL	-	-	66.3 ± 6.5	0.0541	67.5 ± 3.4	0.0087	0.7251
	100 µg/mL	-	-	26.2 ± 4.0	0.0022	38.2 ± 10.4	0.0095	0.1143
LI30 (%)	0 ng/mL	99.8 ± 0.4	99.8 ± 0.4					
	10 ng/mL	-	-	99.7 ± 0.5	>0.9999	99.8 ± 0.4	>0.9999	0.1797
	100 ng/mL	-	-	99.5 ± 0.8	0.7273	99.5 ± 0.8	0.7273	0.0649
	1 µg/mL	-	-	99.6 ± 0.5	>0.9999	99.7 ± 0.5	>0.9999	0.5887
	10 µg/mL	-	-	99.5 ± 0.5	0.5455	100 ± 0.0	>0.9999	0.6991
	100 µg/mL	-	-	68.3 ± 25.3	0.0022	79.5 ± 15.8	0.0048	0.0667

**Table 3:** Unpaired *t*-tests comparing X-fold shift values between venoms with each antivenom.

Antivenom	X-Fold Shift Value for *B. atrox* Venom	X-Fold Shift Value for *B. lanceolatus* Venom	*p*-Value
Bothrofav™	1.92 ± 0.04	1.58 ± 0.08	0.0032
Inoserp^™^ South America	1.398 ± 0.008	0.81 ± 0.05	<0.0001
Antivipmyn^™^ TRI	0.98 ± 0.02	1.25 ± 0.11	0.0124
PoliVal-ICP™	0.84 ± 0.05	0.53 ± 0.08	0.0005

**Table 4:** Antivenoms used in the study.

Antivenom (Manufacturer)	Batch and Expiry Date	Protein Concentration	Immunizing Mixture	Antibodies
Bothrofav^™^ (MicroPharm Limited, Newcastle Emlyn, UK)	P4A561V 10/2020	190 g/L	*B. lanceolatus*	Liquid F(ab’)_2_
Inoserp^™^ South America (Inosan Biopharma, Mexico, Mexico)	0IT06007 06/2022	16.9 g/L	*B. alternatus*, *B. asper*, *B. atrox*, *B. lanceolatus*, *B. diporus*, *B. jararaca*, *B. jararacussu*, *B. schlegeii*, *C. simus*, *L. muta*, *L. melanocephala*, *L. stenophrys*	Freeze-dried F(ab’)_2_
Antvipmyn^™^ TRI (Instituto Bioclon, Mexico, Mexico)	B-7B-32 2022	9 g/L	*B. asper*, *C. durissus*, *L. muta*	Freeze-dried F(ab’)_2_
PoliVal-ICP^™^ (Instituto Clodomiro Picado, Vázquez de Coronado, Costa Rica)	6180219POLQ 02/2022	64.3 g/L	*B. asper*, *C. simus*, *L. stenophrys*	Liquid Whole IgG

*B.: Bothrops, C.: Crotalus, L.: Lachesis*.

**Table 5:** Fibrinogen and coagulation factors of human platelet-poor plasma used in coagulation experiments.

Coagulation Factor	Value
Fibrinogen (g/L)	2.31
Factor II activity (%)	82
Factor V activity (%)	76
Factor VII activity (%)	101
Factor VIII activity (%)	70
Factor IX activity (%)	80
Factor X activity (%)	76
Factor XI activity (%)	93
Factor XII activity (%)	71

## Data Availability

S.L. and B.M. have full access to all data and take responsibility for the data integrity and their analysis accuracy. Data supporting the reported results can be obtained from the corresponding authors if reasonably justified.
